# RPS3 regulates melanoma cell growth and apoptosis by targeting Cyto C/Ca^2+^/MICU1 dependent mitochondrial signaling

**DOI:** 10.18632/oncotarget.4868

**Published:** 2015-08-22

**Authors:** Yun Tian, Lijun Qin, Huijuan Qiu, Dingbo Shi, Rui Sun, Wenbing Li, Tianze Liu, Jingshu Wang, Tingting Xu, Wei Guo, Tiebang Kang, Wenlin Huang, Guowen Wang, Wuguo Deng

**Affiliations:** ^1^ Sun Yat-sen University Cancer Center, State Key Laboratory of Oncology in South China, Collaborative Innovation Center of Cancer Medicine, Guangzhou, China; ^2^ Department of Bone and Soft Tissue Tumors, Tianjin Medical University Cancer Institute and Hospital, National Clinical Research Center for Cancer, Key Laboratory of Cancer Prevention and Therapy, Tianjin, China; ^3^ Department of Pediatrics, Sun Yat-sen Memorial Hospital, Sun Yat-sen University, Guangzhou China; ^4^ Institute of Cancer Stem Cell, Dalian Medical University, Dalian, China; ^5^ State Key Laboratory of Targeted Drug for Tumors of Guangdong Province, Guangzhou Double Bioproduct Inc., Guangzhou, China

**Keywords:** melanoma, RPS3, MICU1, calcium ion, mitochondrial

## Abstract

Melanoma is one of the most aggressive and lethal cancers. Discovery and identification of novel therapeutic targets is urgently needed. In this study, we demonstrated that ribosomal protein S3 (RPS3) was a potential target involved in melanoma growth. Knockdown of RPS3 by siRNA suppressed cell growth and induced apoptosis in melanoma cells. Further mechanism studies showed that RPS3 knockdown in melanoma cells triggered the release of cytochrome C (Cyto C) from mitochondrial, increased the location of BID on mitochondrial membrane and the cleavage of the pro-apoptotic proteins (PARP, caspase-3 and -9), promoted the opening of mitochondrial permeability transition pore and the flooding of calcium ions (Ca^2+^) into the mitochondrial, and decreased the expression of the Ca^2+^ gatekeeper MICU1 and its location on the mitochondrial. We also found that knockdown of RPS3 significantly inhibited tumor growth in a melanoma xenograft mouse model. Furthermore, we showed that RPS3 was highly expressed in melanoma cell lines and melanoma tumor tissues, and overexpression of RPS3 was associated with the poor prognosis of melanoma patients. Our results therefore demonstrate that RPS3 regulates melanoma growth through the modulation of the Cyto C/Ca^2+^/MICU1 dependent mitochondrial signaling and suggest that RPS3 is a potential therapeutic target for melanoma treatment.

## INTRODUCTION

Melanoma is one of the most aggressive and lethal cancers known today [1]. Despite recent advances in therapeutic strategies, melanoma with distant metastasis still portends a poor prognosis with a 5-year survival rate of 16% [2]. Patients diagnosed with melanoma typically have a poor prognosis because of a lack of early symptoms, leading to metastatic disease at the time of diagnosis. The treatment options for melanoma include chemotherapy, surgery, and radiation [3]. Hence, the development of molecular biomarkers that will assist the diagnosis and prognosis of melanoma is urgently needed. An increasing number of diagnostic and prognostic biomarkers for cancer have been identified from genome-wide high-throughput expression profiles [4–7]. These biomarkers provide a valuable platform for personalized medicine and treatment of melanoma patients.

Ribosomal protein S3 (RPS3) is a component of 40S ribosomal subunit, which is important in ribosomal maturation for translational processes [12]. In addition, RPS3 has been shown to have additional extraribosomal functions, such as DNA repair, gene transcription, and apoptosis [13–16]. The RPS3 has been reported to have endonuclease activity, which is increased by protein kinase C delta (PKCδ)-dependent phosphorylation upon exposure to genotoxic stresses [14]. Protein phosphatase 2A (PP2A) regulates the level of phosphorylated RPS3 by interacting with non-ribosomal free RPS3, but not with ribosome-associated RPS3 [15]. Moreover, phosphorylation of RPS3, regulated by Akt, might be a critical factor in determining either proapoptotic function or DNA repair activity in neuronal cells [16]. Furthermore, RPS3 protein is also known to be involved in apoptotic pathway [18–20]. Ribosomal proteins may have apoptotic functions as follows: RPS3 is involved in the apoptotic process in NIH3T3 cells [21]. Another study has been reported that RPS3 is secreted as a homodimer in cancer cells. The increased level of secreted RPS3 was detected in more malignant cells, which were established with continuous exposure of cigarette smoke condensate. These findings suggest that the secreted RPS3 protein is an indicator of malignant tumors [23]. And also, Nagao-Kitamoto H et al [24] showed that RPS3 expression was increased in primary osteosarcoma lesions with lung metastases. The forced expression of RPS3 increased migration and invasion of osteosarcoma cells. All these date indicate that RPS3 plays an important role in cancer progress. However, the precise physiological function of RPS3 and its underlying mechanism involved in melanoma tumorgenesis remains unclear.

Calcium ions (Ca^2+^) are required for the proliferation of mammalian cells [25]. Tumor cells can proliferate in Ca^2+^-deficient media [26]. Transient receptor potential melastatin (TRPM), a calcium ion channel, is particularly important in regulating melanocyte physiology [27]. Several studies have shown that TRPM functions as a tumor suppressor in melanoma cells. Its loss of expression correlates with tumor progression and metastatic potential [28, 29]. Excessive Ca^2+^ influx across the mitochondrial can trigger the permeability transition, leading to cell death [30, 32], and the mitochondrial Ca^2+^ uptake 1 (MICU1) is able to suppress mitochondrial Ca^2+^ uptake [31]. So far, there has been no information about the regulation of RPS3 in the Ca^2+^ /MICU1 dependent mitochondrial signaling.

In this study, we identified RPS3 as a novel melanoma target by siRNA knockdown. We also investigated the role of RPS3 in regulating tumor growth in melanoma cell line and xenograft mouse model and evaluated its clinical significance in melanoma patients. In addition, we also analyzed the effect of RPS3 on the mitochondrial signaling in melanoma cells to elucidate the possible molecular mechanism of RPS3.

## RESULTS

### RPS3 knockdown inhibited cell proliferation and colony formation

**Figure 1:**
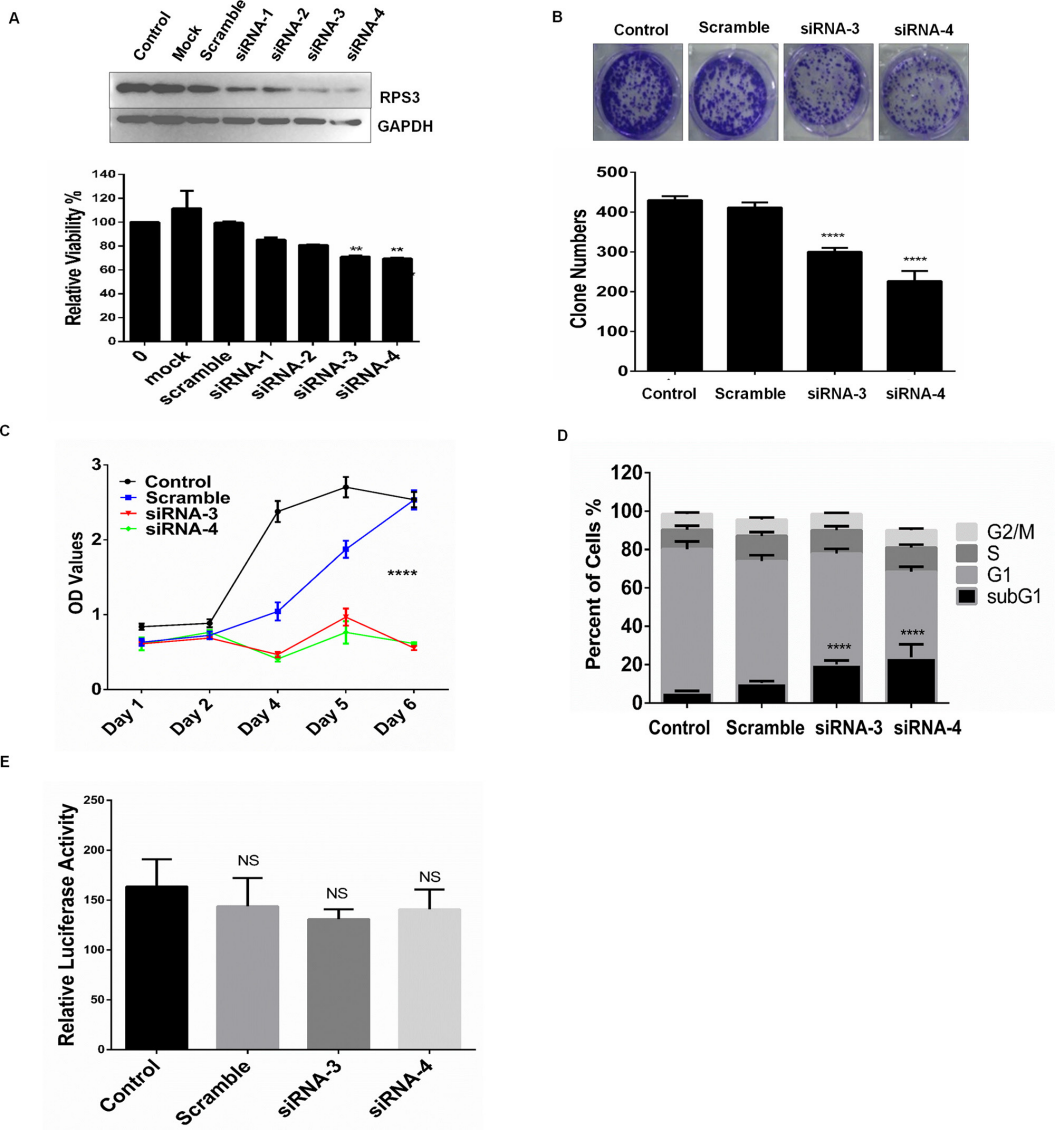
RPS3 knockdown inhibited cell growth **A.** The melanoma A375 cells were treated with 4 siRNAs of RPS3 (50 nM) for 72 hours, and the expression of RPS3 protein and cell viability was analyzed by Western blot and MTS assay, respectively. The mock control and scramble siRNA were used as the control group. **B.** A375 cells were treated with siRNA of RPS3 (50 nM) for 15 days, the numbers of colonies were calculated and statistically analyzed. **C.** A375 cells were treated with siRNA of RPS3 (50 nM) for different days, and cell viability was analyzed by MTS assay and the growth curve were measured. **D.** A375 cells were treated with RPS3 siRNA for 72 hr. The apoptotic sub-G1 cells in cell cycle were detected by propidium iodide single staining and flow cytometry assay. **E.** A375 cells were transfected with a plasmid pRL-CMV following treatment with RPS3 siRNAs. The activity of luciferase was determined to assess the effect of RPS3 on protein translational processes. The data are presented as mean ± S.D. of three separate experiments. *P < 0.05, the significant differences between treatment and control groups.

We quantitatively analyzed the effect of RPS3 knockdown on cell proliferation in A375 cells by MTS assay. As shown in Figure 1A, RPS3 knockdown by transient transfection with four different siRNAs targeting RPS3 (siRNA-1, siRNA-2, siRNA-3 and siRNA-4) markedly inhibited RPS3 protein expression and cell proliferation by comparison with the treatment with the non-specific scramble siRNA (scramble) or mock control in A375 cells. Based on the strong inhibitions of siRNA-3 and siRNA-4 on cell growth, we selected these two siRNAs for the next experiments, which excluded the possibility of the off-target effect of RPS3 siRNA.

We also performed the colony formation experiment to confirm the effect of RPS3 on melanoma cell growth. As shown in Figure 1B, knockdown of RPS3 by siRNA-3 or siRNA-4 effectively inhibited cell clonogenicity, resulting in a marked decrease in colony formation ratio in melanoma A375 cells.

We next investigated the role of RPS3 in the regulation of melanoma growth by plotting the growth curves. The A375 cells were transfected with the RPS3 siRNA-3 and siRNA-4, and the cell viability was measured by MTS every day. As shown in Figure 1C, knockdown of RPS3 by siRNAs significantly suppressed cell viability compared to the non-specific scramble siRNA (*P* < 0.001).

### RPS3 knockdown increased the accumulation of apoptotic sub-G1 cell population

Since the growth inhibitory effect was observed in the siRPS3-transfected melanoma cells, we next analyzed whether RPS3 mediated the accumulation of the apoptotic sub-G1 cell population in A375 cells by flow cytometry. As shown in Figure 1D, knockdown of RPS3 considerably increased the population of the apoptotic sub-G1 phase cells as compared with the groups treated with the non-specific scramble siRNA.

To further clarify that the cell death induced by RPS3 knockdown results from ribosomal malfunction or inhibition of extra-ribosomal function, we next determined the effect of RPS3 on protein translation by a luciferase reporter gene assay. The A375 cells were transfected with a plasmid pRL-CMV, and the activity of luciferase was determined. The results showed that RPS3 knockdown slightly, but not significantly, inhibited the translational processes, suggesting that the cell death was induced mainly by the inhibition of extra-ribosomal function (Figure 1E).

### RPS3 knockdown induced cell apoptosis

**Figure 2:**
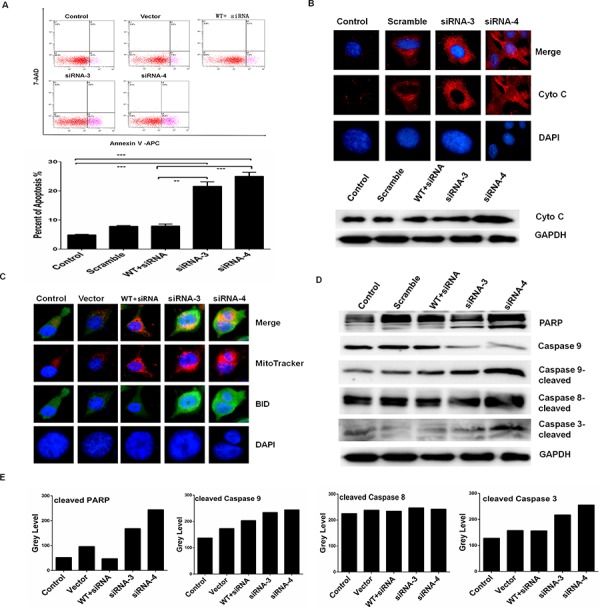
RPS3 knockdown induced apoptosis The melanoma A375 cells were transfected with siRNAs of RPS3 (50 nM) for 72 hours. Apoptosis was tested by annexinV staining and flow cytometry **A.** and the releasing of Cyto-C from mitochondrial and the expression of Cyto-C protein were detected by immunofluorensence staining and Western blot **B.** The pro-apoptotic protein BID translocated to the mitochondrial was determined by immunofluorensence staining **C.** and the levels of the protein markers of apoptosis, such as cleaved PARP, cleaved caspase-9, -8 and -3 were detected by Western blot **D.** and quantitatively analyzed **E.**

Next, we analyzed the effect of RPS3 on apoptosis by flow cytometry. As shown in Figure 2A, knockdown of RPS3 markedly increased the numbers of apoptosis cells (Figure 2A) compared with the scramble siRNA and control group. Conversely, overexpression of RPS3 reversed the RPS3 siRNA 3-induced apoptosis (Figure 2A). Knockdown of RPS3 also triggered the release of cytochrome C (Cyto C) from mitochondrial and up-regulated the expression of Cyto C protein (Figure 2B), and increased the location of BID on mitochondrial membrane (Figure 2C). Moreover, RPS3 knockdown also promoted the cleavage of the pro-apoptotic proteins PARP, caspase-3 and -9 (Figure 2D and 2E).

### RPS3 knockdown promoted mitochondrial transition pore opening and Ca^2+^ flooding

**Figure 3:**
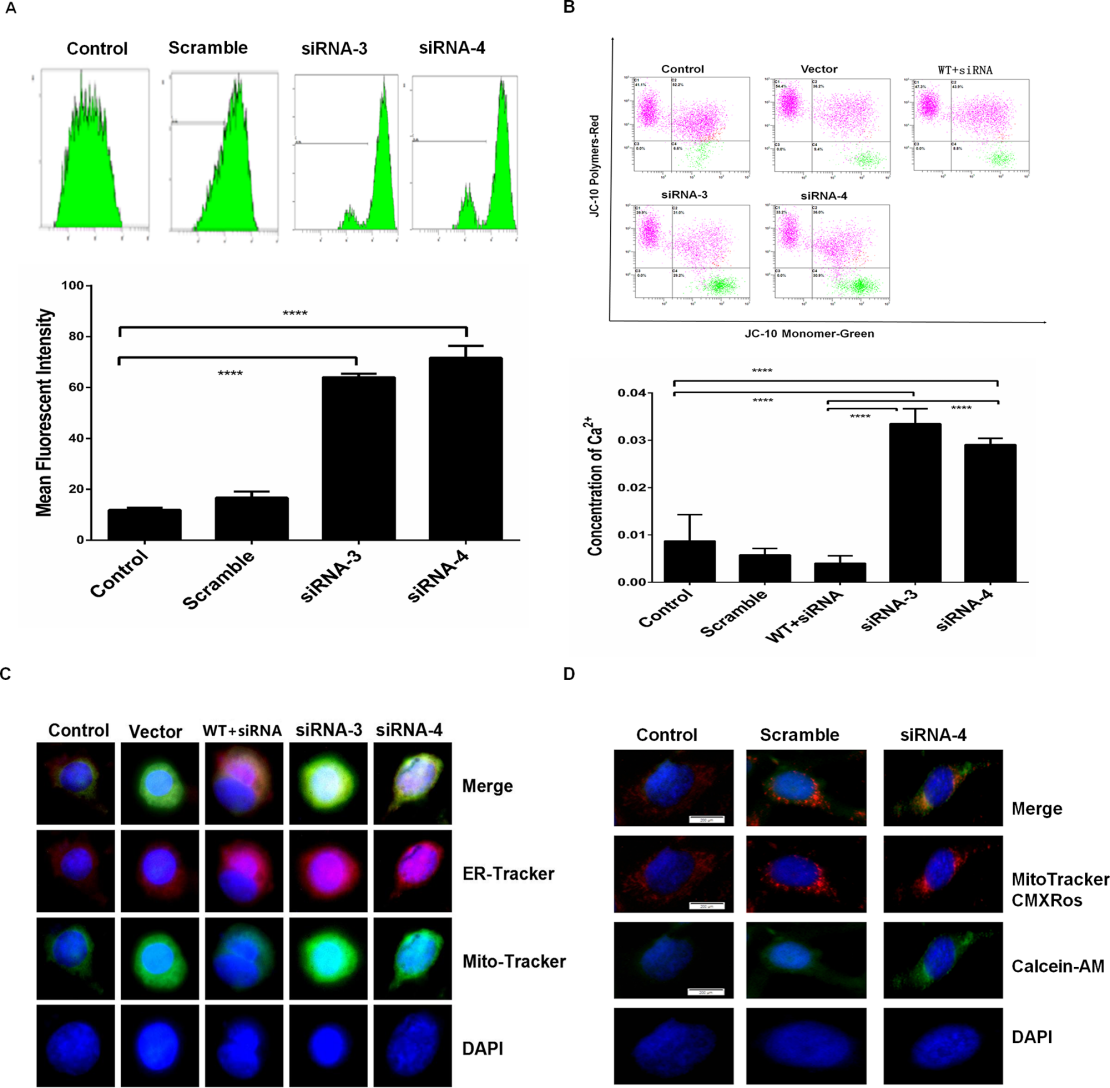
RPS3 knockdown prompted Ca^2+^ flooding into the mitochondrial A375 cells were treated with siRNA of RPS3 (50 nM) for 72 hr. The opening of permeability transition pore **A.** and the membrane potential of mitochondrial depolarization **B.** were determined. The translocation of mitochondrial into the ER **C.** and the flooding of calcium ions flooding into the mitochondrial **D.** were also analyzed by confocus microscope.

**Figure 4:**
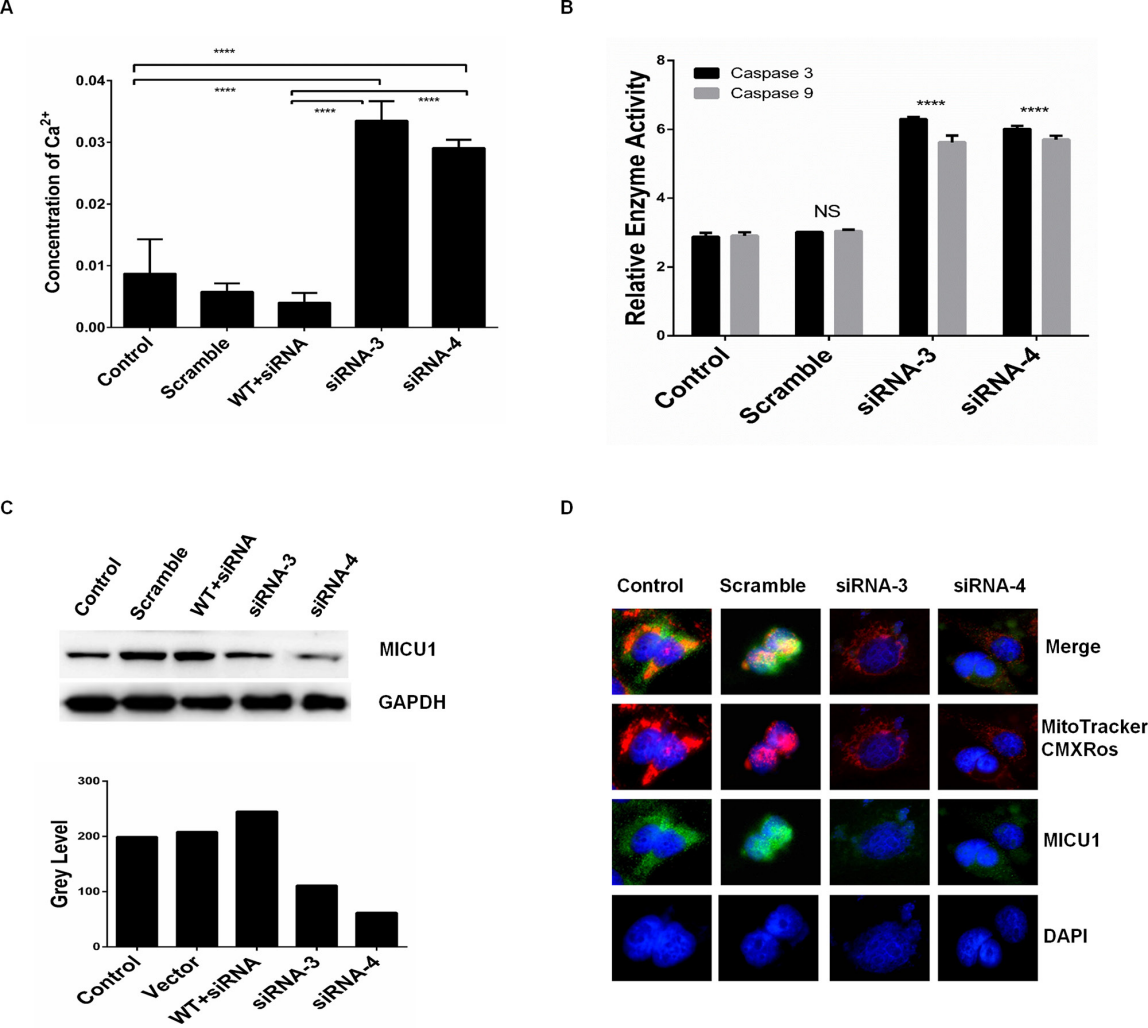
RPS3 knockdown reduced the mitochondrial Ca^2+^ gatekeeper MICU1 A375 cells were treated with siRNA of RPS3 (50 nM) for 72 hr. The concentration of calcium ions in cells **A.** and the activities of caspas-3 and -9 **B.** were determined. The expression of mitochondrial Ca^2+^ gate keeper protein MICU1 **C.** and its co-localization with MitoTrackerCMXRos **D.** were examined by Western blot and confocus microscope, respectively.

To identify the possible mechanisms of RPS3 in melanoma cells, we next detected the effects of RPS3 knockdown on mitochondrial transition pore status and Ca^2+^ dependent signaling. As compared with the scramble and control group, RPS3 knockdown markedly induced the opening of mitochondrial transition pore (Figure 3A). Paralleled with the mitochondrial permeability transition pore opening, knockdown of RPS3 increased the mitochondrial membrane potential (Figure 3B). Conversely, overexpression of RPS3 decreased the mitochondrial membrane potential (Figure 4B). We also found that RPS3 knockdown promoted mitochondrial translocation to the ER by staining with the mitochondrial and ER specific probes Mito-Tracker and ER-Tracker (Figure 3C), and increased calcium ions (Ca^2+^) entering into the mitochondrial by staining with the mitochondrial specific probe MitoTrackerCMXRos (Figure 3D).

### RPS3 knockdown decreased MICU1 expression and its location on the mitochondrial

We further confirmed the role of RPS3 in the regulation of mitochondrial calcium ions (Ca^2+^) in melanoma cells. RPS3 knockdown obviously increased the concentration of Ca^2+^, and overexpression of the wild type RPS3 significantly reduced the raise of Ca^2+^ (Figure 4A). Paralleled with the increase of Ca^2+^, RPS3 knockdown significant increased the activity of caspase 3 and caspase 9 in A375 cells (Figure 4B). In addition, knockdown of RPS3 markedly decreased the expression of MICU1, a key regulator of mitochondrial calcium ions (Ca^2+^), in the whole cell lysate (Figure 4C). In the control group, MICU1 was located on the mitochondrial, however, when RPS3 was knocked down by siRNA, the location of MICU1 on the mitochondrial was significantly decreased (Figure 4D).

### RPS3 knockdown inhibited tumor growth in a xenograft mouse model *in vivo*

**Figure 5:**
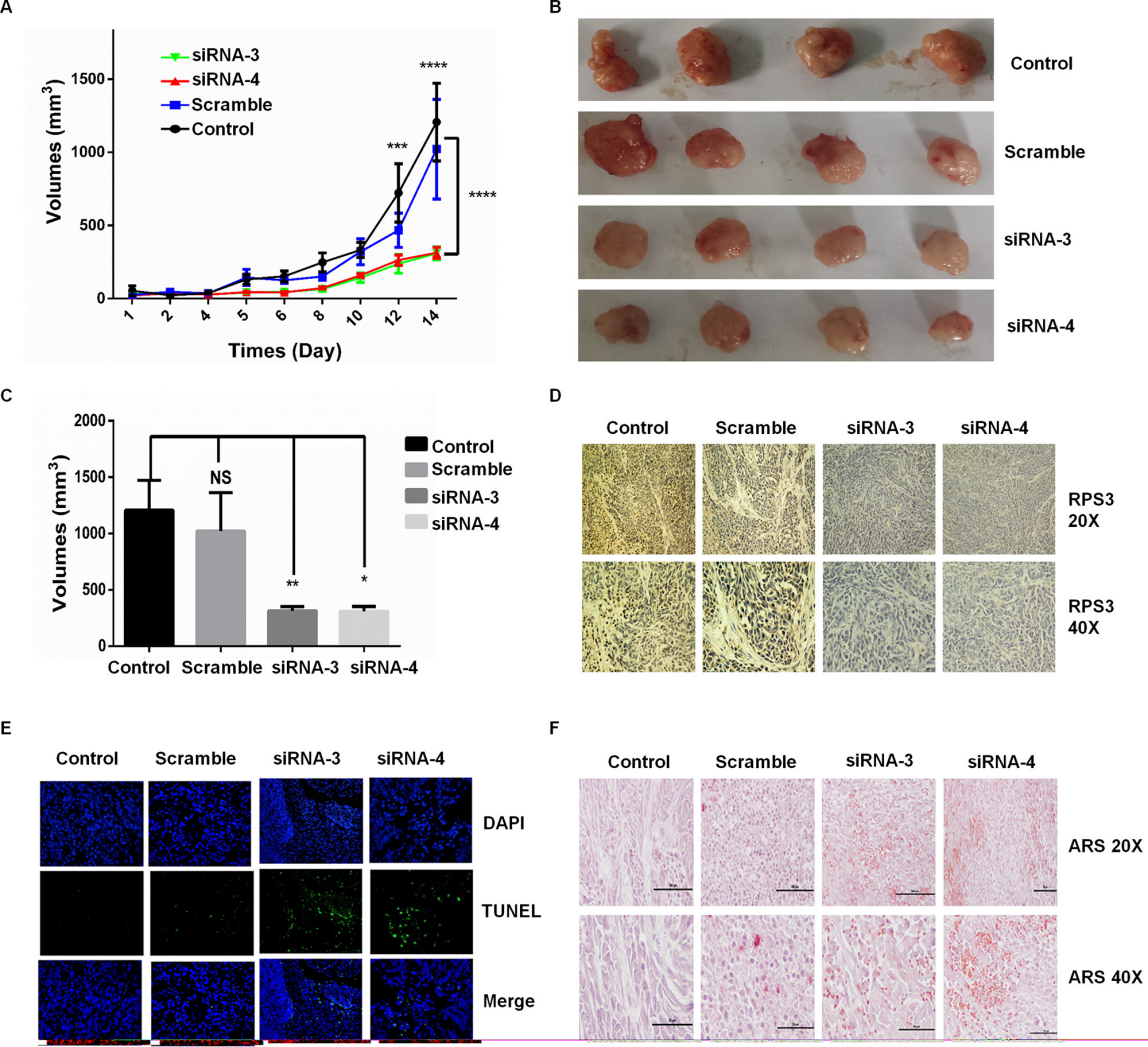
Knockdown of RPS3 inhibited tumor growth on melanoma *in vivo* Melanoma A375 cells were injected subcutaneously into the flank of nude mice, and the visible tumors developed at the injection sites after 4 days. The DC nanoparticle-encapsulated RPS3 siRNA was then intratumorally injected twice per week. The effect of RPS3 siRNA on tumor growth curves were analyzed based on the volume at the indicated days **A.** At the 14^th^ day, the tumors **B.** and their volumes **C.** were compared. The expression of RPS3 protein **D.** and the apoptosis in tumor tissues **E.** was analyzed by IHC and TUNEL assays. The ARS staining was used to analyze the concentration of Ca^2+^ in tumor tissues **F.** Data is tumor volumes ± SE in nude mice, *n* = 5.

To confirm the roles of RPS3 in the regulation of melanoma growth and survival, we analyzed the effect of RPS3 on tumorigenicity *in vivo* using a xenograft mouse model. A375 cells were injected subcutaneously into the flank of nude mice, and the visible tumors developed at the injection sites after 4 days. The DC nanoparticle-encapsulated RPS3 siRNA was then intratumorally injected twice per week for three weeks. Knockdown of RPS3 significantly suppressed tumor growth (Figure 5A, 5B and 5C) and RPS3 protein expression (Figure 5D) compared to the treatment with the non-specific scramble siRNA. We also analyzed the effect of RPS3 knockdown on apoptosis in tumor tissues by TUNEL assay. As shown in Figure 5E, treatment with RPS3 siRNA effectively induced apoptosis. Moreover, alizarin red (ARS) staining in tumor tissues showed that knockdown of RPS3 effectively increased the Ca^2+^ red dots by comparison with the scrimble or control group (Figure 5F).

### High expression of RPS3 predicted a poor prognosis in melanoma patients

**Figure 6:**
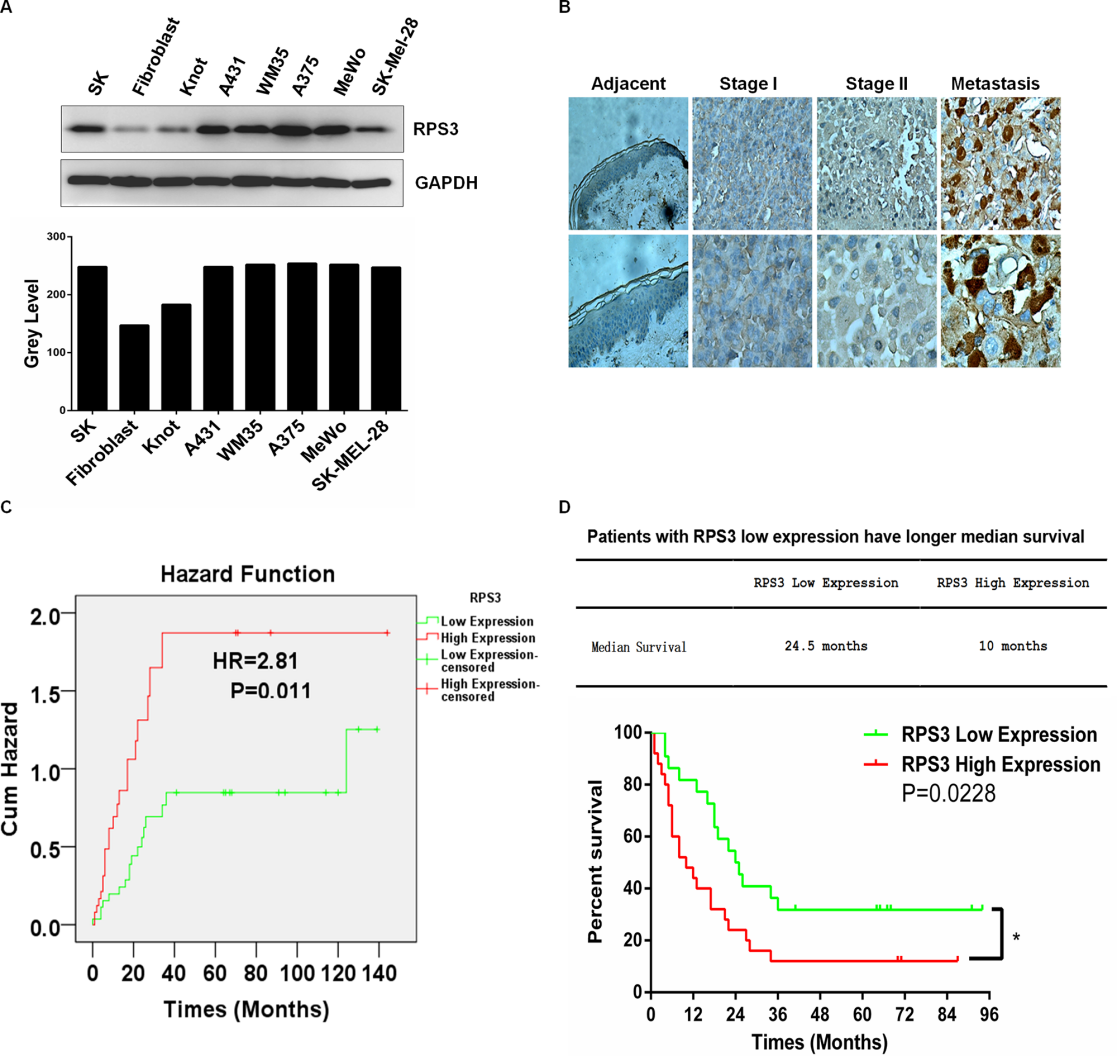
RPS3 was highly expressed in melanoma cell lines and tumor tissues and associated with poor prognosis **A.** The expression of RPS3 in human melanoma cell lines was analyzed by Western blot. **B.** The expression of RPS3 in melanoma tumor tissues was detected by immunohistochemical staining. (magnification, × 400). **C.** Patients with high levels of RPS3 had high risk than those who with low levels of RPS3. (Hazards ratio HR = 2.81, *P* = 0.011). **D.** Melanoma patients with low RPS3 expression had longer median survival times than those with high RPS3 expression (*p* = 0.0228, *n* = 59).

We evaluated the association of RPS3 expression with the melanoma patient survival and clinical outcome. We first examined the expression of RPS3 at protein levels in five human melanoma cell lines (A375, A431, WM35, MeWo, SK-MEL-28), one immortalized normal human cell SK and two normal cell lines (fibroblast, Knot) by Western blot analysis. The RPS3 protein levels were up-regulated in all five melanoma cell lines and the immortalized cell (SK) by comparison with the RPS3 expression in two normal cell line (fibroblast, Knot) (Figure 6A). To further confirm the high expression of RPS3 in melanoma and to investigate the clinicopathological significance of RPS3 expression, we also analyzed the expression of RPS3 in tumor tissues from 60 cases of patients with melanoma by immunohistochemical staining assay. Consistent with the results from Western blot analysis, the high levels of RPS3 proteins were detected in melanoma tumor tissues. The RPS3-positive staining was observed in melanoma cells of tumor tissues, but the staining was negative in the adjacent non-tumor tissue samples surrounding tumors (Figure 6B). These results suggest that high level of RPS3 may be a potential target for melanoma.

We also analyzed the relationship of hazards ratio and survival time with RPS3 protein expression in melanoma patients. Compared with the patients who expressed low levels of RPS3, the patients with high levels of RPS3 had more risks. The hazards ratio (HR) was 2.81 (*P* = 0.011) (Figure 6C). The median survival time of the melanoma patients with low expression of RPS3 was 24.5 months, whereas the median survival time of the melanoma patients with high expression of RPS3 was 10 months (Figure 6D). Also, the overall survival in the low RPS3 expression patients were significantly improved compared to the high RPS3 expression patients. High RPS3 expression patient group had significantly low overall survival time than the patient group with low RPS3 expression (*P* = 0.0228) (Figure 6D).

## DISCUSSION

In this study, we identified RPS3 as a new melanoma target. The prognosis of patients with high levels of RPS3 was worse than those with low RPS3 levels. We also showed that knockdown of RPS3 could induce the opening of mitochondrial permeability transition pore, and this opening caused the remarkable release of cytochrome C (Cyto C) from mitochondrial to the cytoplasm, and finally induced apoptosis. In this process, Ca^2+^ was found to flood into the mitochondrial. RPS3 knockdown also inhibited the mitochondrial Ca^2+^ gatekeeper MICU1 and its location on mitochondrial.

Calcium ion (Ca^2+^) is an important second messager, involved in many important physiology activities. Although cancer cells can proliferate in Ca^2+^ deficient media [26], calcium ions are still used to stimulate cancer cell to proliferation. Previous study has shown that transient receptor potential melastatin (TRPM) is expressed on melanoma cells, and the member TRPM2 is conferring susceptibility to cell death upon oxidative stress [33]. In contrast, TRPM7 has a protective and detoxifying function in melanoma cells, and it is highly expressed in metastatic melanoma [34]. One study propose that functional relevance for Ca^2+^-driven survival of melanoma cells due to the control of store-operated Ca^2+^ entry (SOCE) by the mitochondrial [35]. Uptake of Ca^2+^ by mitochondrial has been known long time ago, and excessive uptake of Ca^2+^ was harmful to cells, but the underlying molecular mechanisms remain unclear. Fabiana Perocchi et al [36] discovered that mitochondrial calcium uptake 1 (MICU1) did not abolish mitochondrial respiration or membrane potential, but activated matrix dehydrogenases. In this study, we also found that knockdown of RPS3 promoted the opening of mitochondrial transition pore, thereby induced Ca^2+^ obviously influx to the mitochondrial, but this influx obviously is too much. Instead of stimulating melanoma cells proliferation, the RPS3 knockdown-mediated flooding of Ca^2+^ into mitochondrial induced apoptosis.

Our study also found that RPS3 knockdown downregulated MICU1 and induced Ca^2+^ flooding into the mitochondrial. Over expression of RPS3 could reverse the decrease of MICU1. Based on these data, we infer that RPS3 promotes the stability of MICU1, and thus prevents the Ca^2+^ influx. The co-localization data also indicated that RPS3 knockdown decreased MICU1 location on mitochondrial. This might imply that RPS3 also fixed MICU1 to the mitochondrial, and by knocking down RPS3, MICU1 could not locate on mitochondrial. However, whether RPS3 fixed MICU1 to the mitochondrial and how it stabilized MICU1 need further study.

In summary, we discovered and identified ribosomal protein S3 (RPS3) was a potential molecular target for melanoma. Knockdown of RPS3 by siRNA suppressed cell growth and induced apoptosis in melanoma cells. We also found that RPS3 knockdown promoted the opening of mitochondrial permeability transition pore, triggered mitochondrial membrane potential depolarized and the flooding of calcium ions (Ca^2+^) into the mitochondrial, and decreased the level of the Ca^2+^ gatekeeper MICU1 and its location on the mitochondrial in melanoma cells. Furthermore, we showed that RPS3 was highly expressed in various melanoma cell lines and melanoma tumor tissues and the overexpression of RPS3 was associated with the poor prognosis of patients. Our results therefore demonstrated that RPS3 regulated melanoma growth through the modulation of the Ca^2+^/MICU1 dependent mitochondrial signaling and suggest that RPS3 is a potential therapeutic target for melanoma treatment.

## MATERIALS AND METHODS

### Cell culture

The human melanoma cell lines A375, A431, WM35, MeWo and SK-mel-28 were obtained from the American Type Culture Collection (ATCC). The normal fibroblasts were kindly provided by Dr. Bin Tang at the First Affiliated Hospital of Sun Yat-sen University. Cells were grown in DMEM supplemented with 10% FBS, 100 IU/mL penicillin G and 100 μg/mL streptomycin. The immortalized human melanoma epithelial cell line Knot and SK was kindly provided by Dr. Tiebang Kang at Sun Yat-sen University Cancer Center. Knot and SK cells were maintained in keratinocyte serum-free medium supplemented with epidermal growth factor (0.2 ng/mL) and bovine pituitary extract (30 μg/mL). All media reagents were obtained from Invitrogen. The cell lines were routinely maintained at 37°C in a humidified 5% CO_2_ atmosphere.

### Transfection

Transfection was performed using Lipofectamine 2000 (Life Technologies) according to the manufacturer's recommendations. Cell culture medium was replaced 24 h post-transfection and cells were allowed to grow for additional 72 h before any treatment.

### MTS assay

The cells were seeded into 96-well plates at a concentration of 8 × 10^3^ cells/well. The reagents MTS (Promega, USA) was added to the wells at 72 h post-transfection, and cells were diluted in normal culture medium at 37°C. The absorbance values in each well were measured with a microplate reader set at 490 nm. All experiments were performed three times and the average percentages of cells are shown.

### Apoptosis assay

Apoptosis detection was performed using flow cytometry. The A375 cells were transfect with siRNA and wild type RPS3 plasmid. After 3 days, cells were collected and stained with AnnexinV-APC and 7-AAD.

### Mitochondrial membrane potential assay

The A375 cells were transfect with siRNA and wild type RPS3 plasmid. After 3 days, cells were collected and incubated with JC-10 at 37C, 5% CO_2_ for 20 minutes. The signals were detected through flow cytometry.

### Alizarin red staining

To evaluate the calcium ions concentration in tissues, we performed the alizarin red staining and watch under microscope. The positive cells were stained as nacarat dots.

### Caspase 3 and caspase 9 activity assay

The activity of caspase-3 or caspase-9 was detected using the kits according to the instruction.

### Luciferase reporter gene assay

The A375 cells were treated with pRL-CMV. Three days later, the luciferase activity was detected at Spectramax M5.

### Western blot analysis

Cells were transfected with 20 nmol/L of RPS3 siRNA or non-silencing siRNA in 6-well plates. Whole cell lysates were prepared by using Complete Lysis-M reagent (Roche). Protein concentration was determined by BCA assay (Pierce) and lysates were resolved by SDS-PAGE on a 10% to 12% resolving gel. Proteins were transferred onto polyvinylidene difluoride membranes. Antibodies for RPS3, BID, glyceraldehyde-3-phosphate dehydrogenase (GAPDH), caspase8, PARP, caspase9, caspase 3, MICU1 were purchased from Cell Signaling Technology.

### Mitochondrial transition pore assay

To evaluate the permeability state of the mitochondrial, we use the calcium ions probe calcein-AM and the specific quencher CoCl2. Cells were digested by typsin without EDTA and re-suspend by HBSS/Ca. Calcein-AM and CoCl_2_ were added to the cell suspensions and incubated at 37°C for 15 mins, and then 3.5 ml HBSS/Ca (3.5 ml) was added following centrifuge at 600 g for 5 mins. The cells were resuspended by 500 μl 0.9% sodium chloride and detected at flow cytometry.

### Immunofluorescence

To dectect the distribution of MICU1 and calcium ions, immunofluorescence was used. Melanoma cells were fixed by 4% paraformaldehyde, perforate by 2% Triton X-100 and added the corresponding primary antibodies or probes. The images were watched under fluorescence microscope.

### TUNEL assay

To evaluate the apoptosis in tumor tissues, we made the pathological slice. The slides were dewaxed by xylene and fixed by 4% paraformaldehyde and permeability by Protease K. Mix enzyme and label solution, add to the slice and incubate at 37°C for 60mins. Mount on the slice and watch under fluorescence microscope.

### Animal experiments

To determine the effect of RPS3 on melanoma tumor growth in a xenograft model, six week old athymic Nu/nu nude mice were maintained in a pathogen-free environment. Ten NC and ten RPS3 knock-down mice groups were used (total of 20 mice). In brief, A375 cells (2 × 10^6^) were inoculated subcutaneously into the flank of the nude mice. Once palpable tumors developed (Average volume 50 mm3), mice tumors were treated with 100 nM synthetic RPS3 siRNA complexed with 100 μl transfection reagent in 50 μl PBS was delivered nine times intratumorally every three days. Tumor growth was followed for 14 days from first injection until tumors reached about 400 mm3 in total volumes, at which time mice were euthanized. The tumor volume was calculated using the following formula: Volume = π × Length × Width × High /6. Body weights were also recorded. Primary analyses involved the comparison (for each time point separately) between the NC group and the RPS3 siRNA group. Animal experiments were approved by the Animal Research Committee of Sun Yat Sen University cancer center and were performed in accordance with established guidelines.

### Statistical analysis

Data were expressed as the mean ± standard deviation of at least three independent experiments. Statistical analysis was carried out using SPSS 11.0 software (SPSS Inc.; Chicago, IL, USA). *P* values < 0.05 were considered significant.
